# Population and Landscape Genetics of an Introduced Species (*M. fascicularis*) on the Island of Mauritius

**DOI:** 10.1371/journal.pone.0053001

**Published:** 2013-01-14

**Authors:** Jessica Satkoski Trask, Debra George, Paul Houghton, Sree Kanthaswamy, David Glenn Smith

**Affiliations:** 1 Department of Anthropology, University of California Davis, Davis, California, United States of America; 2 California National Primate Research Center, University of California Davis, Davis, California, United States of America; 3 Primate Products, Inc., Immokalee, Florida, United States of America; 4 Department of Environmental Toxicology, University of California Davis, Davis, California, United States of America; Instituto de Higiene e Medicina Tropical, Portugal

## Abstract

The cynomolgus macaque, *Macaca* fascicularis, was introduced onto the island of Mauritius in the early 17^th^ century. The species experienced explosive population growth, and currently exists at high population densities. Anecdotes collected from nonhuman primate trappers on the island of Mauritius allege that animals from the northern portion of the island are larger in body size than and superior in condition to their conspecifics in the south. Although previous genetic studies have reported Mauritian cynomolgus macaques to be panmictic, the individuals included in these studies were either from the southern/central or an unknown portion of the island. In this study, we sampled individuals broadly throughout the entire island of Mauritius and used spatial principle component analysis to measure the fine-scale correlation between geographic and genetic distance in this population. A stronger correlation between geographic and genetic distance was found among animals in the north than in those in the southern and central portions of the island. We found no difference in body weight between the two groups, despite anecdotal evidence to the contrary. We hypothesize that the increased genetic structure among populations in the north is related to a reduction in dispersal distance brought about by human habitation and tourist infrastructure, but too recent to have produced true genetic differentiation.

## Introduction

Cynomolgus macaques, *Macaca fascicularis*, are distributed widely throughout mainland Indochina and insular Southeast Asia [1]. They are also found in large numbers on the island of Mauritius, where they are an invasive species. Monkeys were likely transplanted to Mauritius from the Indonesian islands [2], [3] by Dutch or Portuguese sailors in the early 1600's, along with other invasive species, such as pigs, sheep and rats [4].

The island of Mauritius encompasses a total area of only 1860 square kilometers and population estimates of cynomolgus macaques have ranged from as high as 35,000 to 40,000 animals in the 1980's [4] to as low as 8,000 animals currently in the wild [5]. Population censuses conducted in 2006 and 2010 indicate that that the wild population has remained steady at 30,000–40,000, and animals in captivity at two breeding centers number an additional 40,000 individuals.

Although the rhesus macaque (*Macaca mulatta*) is still the most commonly used non-human primate research model, the number of cynomolgus macaques imported into the United States for research purposes is increasing rapidly, with 126,000 cynomolgus macaques imported into the United states between the years of 2000 and 2005 [6].

In FY2009, 19% of all cynomolgus macaques imported into the United States originated in captive breeding centers on Mauritius [7]. Mauritian individuals are naturally free of viruses such as measles, Herpes B and and SRV-1 and -2 [8]. The genetic homogeneity of the major histocompatibility complex of the Mauritian cynomolgus macaque is greater than that of other populations of cynomolgus macaques in Southeast Asia, allowing researchers to assemble cohorts of MHC-identical individuals for infectious disease research [9]. The ability to control for the influence of MHC haplotypes on infectious disease susceptibility has contributed significantly to the popularity of Mauritian-origin cynomolgus macaques in biomedical research.

The assumed genetic homogeneity of Mauritian cynomolgus macaques at other loci also contributes to the preferential inclusion of these animals in other biomedical research protocols. Several previous studies have examined the genetic composition of Mauritian cynomolgus macaques based on electrophoretically defined blood proteins polymorphisms [10], microsatellites [11], [12], mtDNA [3], [13]–[15] and Y chromosome lineages [2], [11] and a variety of MHC class I and II alleles [16]–[18]. MtDNA and MHC-based studies have found uniformly low levels of genetic diversity, while Kawamoto's [11] study of 10 microsatellite loci reported little differentiation among sampling sites and concluded that populations on the island were probably panmictic. It is commonly assumed that the observed genetic homogeneity of Mauritian cynomolgus macaques is due to a sudden population expansion from a small number of founder animals. However, the majority of these studies employed individuals from captive colonies whose geographic provenience on Mauritius is unknown. Kondo [10] and Kawamoto [11] collected samples from sites on the southern coast of Mauritius, but the genetic characteristics of animals known to have originated in the northern and central parts of the island have not been examined. Thus, it is possible that the low level of genetic variation reported for Mauritian cynomolgus macaques reflects biased sampling in previous studies.

Anecdotal reports of trappers employed by breeding farms and biomedical research companies on Mauritius allege that animals from the northern half of the island can be easily distinguished from those in the south, with the northern animals exhibiting both larger body size and better condition than those in the south. While neither such a difference, nor its causes (whether environmental or genetic), has been quantitatively documented, the larger animals may be gaining a nutritional advantage from human presence. Muruthi et al. [19] found that female baboons (*Papio* cynocephalus) at Amboseli National Park in Kenya that fed from a lodge garbage dump were just as well nourished as, but spent half as much time feeding and had a much smaller home range than, other animals [20]. The affinity of cynomolgus macaques for human garbage as a food source is also well documented [5], [21], [22]. As early as 1973, Kurland [23] reported that macaques in the Kutai Reserve, Indonesia were disproportionately attracted to human habitation, specifically due to the availability of garbage. More recently, Sha et al. determined that at least 50% of the cynomolgus macaques in Singapore obtain at least some of their diet from anthropogenic sources [24]. Richard et al. [21] described cynomolgus macaques as a “weed species” due to the important roles that garbage and crop raiding play in their feeding ecology in all areas where their range overlaps human habitation.

**Figure 1:**
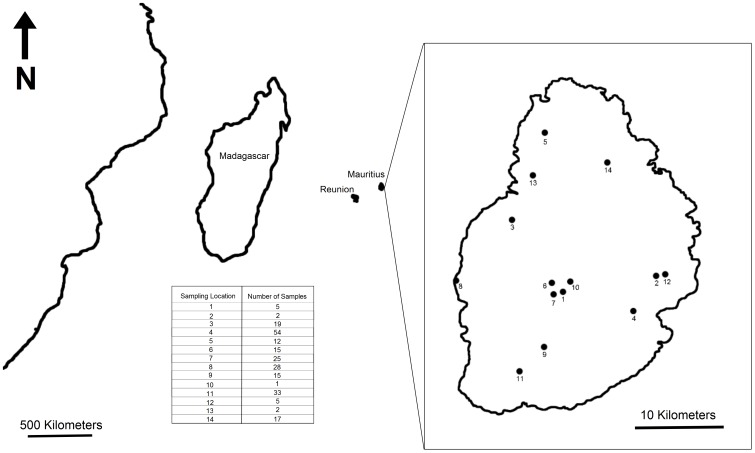
Geographic positions and number of samples collected from each of 14 of sampling sites.

We collected 233 wild cynomolgus macaques from 14 different sites on the island of Mauritius (locations and sample numbers shown in Figure 1) and recorded their age and weight upon capture. We genotyped these animals at 28 microsatellite loci previously optimized for this species [25], [26]. The present study expands on the earlier studies by broadening the scope of sampling into the central and northern regions of Mauritius, where cynomolgus macaques have not been genetically characterized, and doubling the number of microsatellite loci examined. The broader distribution of samples allows examination of genetic diversity relative to island features such as topography, urban development and human population density. We also quantitatively examine the anecdotal reports of differences in weight relative to geography.

We found low genetic diversity in Mauritian cynomolgus macaques compared to other regional populations of this species, and no significant genetic or phenotypic (e.g., weight at capture) differentiation with regard to geography. However, a spatial principle component (sPCA) analysis found that the correlation between genetic differentiation and geographic distance was greater among populations on the northern coast of the island than elsewhere. Given the rapid population expansion and high population density of cynomolgus macaques on Mauritius, we discuss these results in relation to topography, land use and human population density.

## Results

Estimates of heterozygosity and allele numbers for each sampling site can be found in Table S1. The total number of observed alleles and observed and expected estimates of heterozygosity are shown in Table 1. The average number of alleles per locus when all sites were pooled was 6.14 ±2.88. The difference between observed and expected heterozygosity was tested via comparison to a chi-square distribution and Holm's sequential Bonferroni method [27] was used to correct for multiple comparisons. Corrected p values of these comparisons are also shown in Table 1.

**Table 1:** The total number of observed alleles and observed and expected heterozygosity in the northern and southern clusters, as well as pooled across all sites.

	All Sites Pooled				Northern Sites				South/Central Sites			
Locus Name	Alleles	H_E_	H_O_	P value	Alleles	H_E_	H_O_	P value	Alleles	H_E_	H_O_	P value
D1s548	3	0.475	0.472	1.00	3	0.428	0.429	0.28	3	0.489	0.486	1.00
D5s1457	3	0.501	0.526	1.00	3	0.553	0.509	1.00	3	0.482	0.531	1.00
D6s501	6	0.601	0.558	1.00	6	0.573	0.526	1.00	6	0.607	0.568	1.00
D7s794	7	0.694	0.674	1.00	6	0.644	0.649	1.00	7	0.708	0.682	1.00
D7s1826	7	**0.596**	**0.575**	0.00	5	0.564	0.545	1.00	7	**0.598**	**0.584**	0.00
D8s1106	6	0.742	0.685	0.34	4	0.713	0.772	1.00	6	0.750	0.657	0.29
D8s1466	5	0.462	0.441	1.00	5	0.415	0.463	1.00	5	0.475	0.434	1.00
D9s921	6	0.810	0.702	0.22	6	0.818	0.815	1.00	6	**0.801**	**0.667**	0.01
D13s765	5	0.593	0.478	0.46	4	0.607	0.545	1.00	5	0.578	0.456	0.38
D3s1768	9	**0.766**	**0.211**	0.00	6	**0.778**	**0.250**	0.00	8	**0.730**	**0.199**	0.00
D4s1626	5	0.645	0.661	1.00	5	0.671	0.714	1.00	5	0.632	0.643	1.00
D4s2365	6	0.659	0.603	1.00	4	0.667	0.571	1.00	6	0.657	0.613	1.00
D11s1975	4	0.654	0.534	0.06	3	0.654	0.544	1.00	4	0.650	0.531	0.31
D11s2002	6	0.665	0.626	1.00	6	0.626	0.527	1.00	6	0.675	0.659	1.00
27Oe8	5	0.681	0.691	1.00	5	0.677	0.614	1.00	5	0.680	0.716	1.00
270o7	4	**0.694**	**0.478**	0.00	4	0.664	0.464	0.34	4	**0.698**	**0.483**	0.00
271j8	8	**0.816**	**0.765**	0.00	7	0.822	0.709	0.14	8	0.806	0.784	0.32
D2s2952	12	**0.796**	**0.396**	0.00	9	**0.822**	**0.393**	0.00	11	**0.776**	**0.397**	0.00
D10s1432	5	0.334	0.289	0.34	5	0.237	0.204	1.00	4	0.360	0.316	0.09
AGAT007	6	0.709	0.730	1.00	5	0.724	0.649	1.00	6	0.698	0.757	1.00
D9s934	6	0.731	0.720	1.00	6	0.695	0.702	1.00	6	0.739	0.726	1.00
D19s255	4	**0.428**	**0.000**	0.00	2	**0.459**	**0.000**	0.00	4	**0.301**	**0.000**	0.00
D14s306	13	**0.827**	**0.678**	0.00	10	0.835	0.712	0.10	12	0.818	0.667	0.41
D16s750	2	0.500	0.500	1.00	2	0.499	0.474	1.00	2	0.500	0.509	1.00
272o12	4	0.294	0.288	1.00	4	0.340	0.327	1.00	4	0.277	0.275	1.00
267o11	14	0.818	0.733	0.60	12	0.818	0.691	1.00	13	0.813	0.747	0.12
D18s537	4	**0.657**	**0.376**	0.00	3	**0.650**	**0.389**	0.00	4	**0.657**	**0.371**	0.00
D18s536	7	**0.318**	**0.291**	0.00	5	0.395	0.439	1.00	6	**0.290**	**0.241**	0.00
**Average**	**6.13**	**0.623**	**0.524**		**5.18**	**0.620**	**0.522**		**5.93**	**0.616**	**0.525**	

Loci significantly out of Hardy-Weinberg equilibrium are shown in boldface.

**Figure 2:**
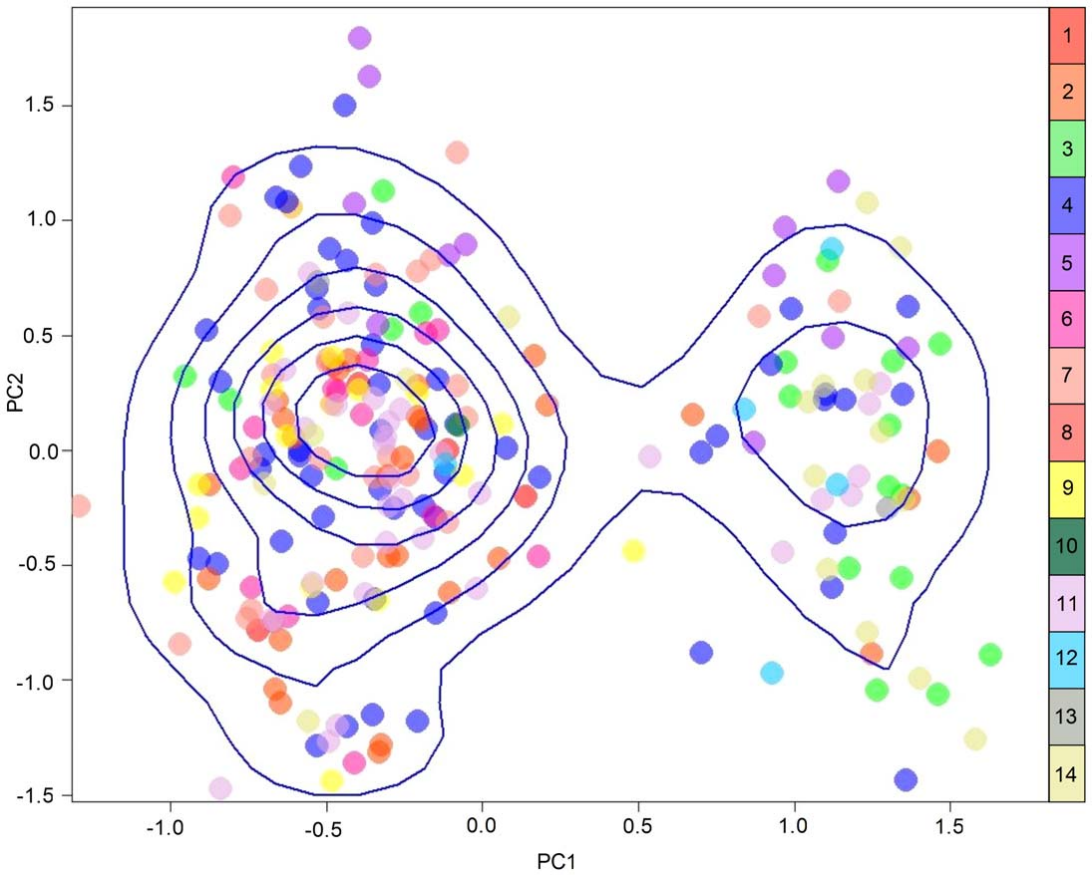
A principle component analysis of all sampled individuals, color-coded by sampling site. The blue lines illustrate the three-dimensional density of individuals in the plot, calculated via kernel density estimate. This spatial analysis demonstrates the presence of two distinct clusters within the data, but the clusters are unrelated to the geographic arrangement of samples.

Figure 2 shows the principle component analysis (PCA) with the individuals grouped by sampling site with a kernel density estimate of the genotypes superimposed over the distribution of individuals. Although there was clearly no genetic differentiation among the sampling sites, Figure 2 indicates that there are two moderately differentiated clusters of animals independent of sampling site (although perhaps not of geographic location), as well as a few individual outliers. Very similar allele frequencies at neighboring sampling sites produced a positive spatial autocorrelation, or global structure, while neighboring sites with very different allele frequencies produce a negative spatial autocorrelation, or local structure. The sPCA produced a total of six global eigenvalues and six local eigenvalues (data not shown). These genetic data are characterized by low variance (indicated by a small total number of eigenvalues), only one local structure of sufficient value to be clearly differentiated from the other local eigenvalues and two global structures large enough to be discernible. The two moderately differentiated clusters shown in Figure 2 are the likely source of the two global eigenvalues.

**Figure 3:**
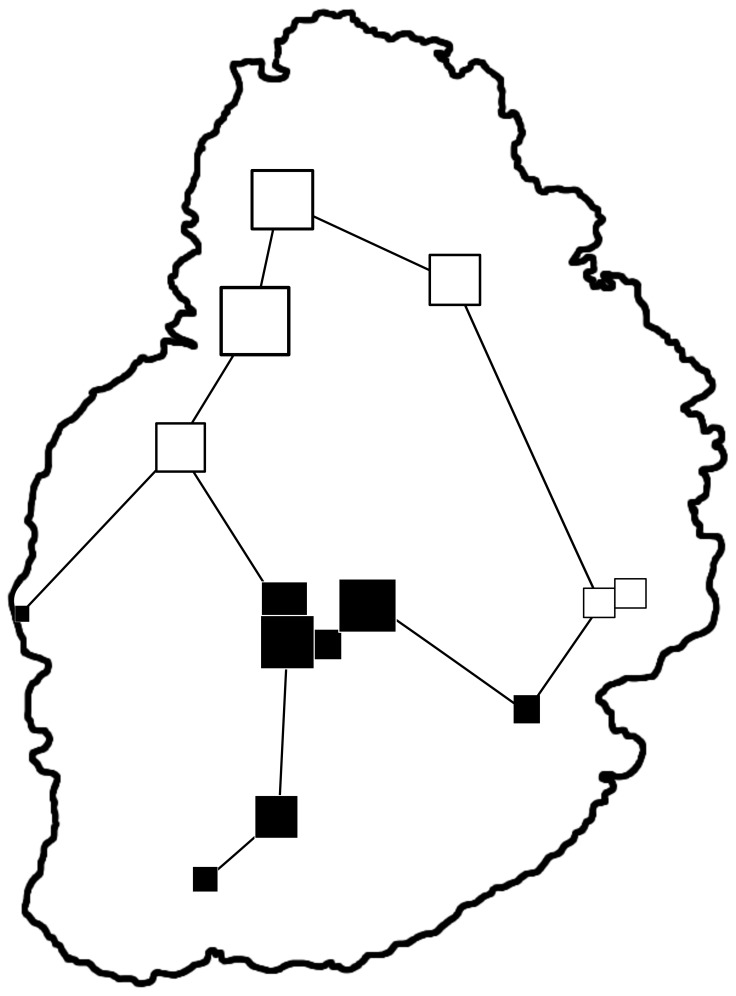
The nearest-neighbor connection network used to define spatial relationships between sampling sites. The size and color of the square is relative to the global scores of the spatial autocorrelation analysis; for example, large white squares denote a large, negative global score and large, black squares denote large, positive global scores.

The results of the spatial principle component analysis (sPCA) are illustrated in Figure 3. The connection network used to define spatial weightings for the relative neighbor method is illustrated with dark lines, while the squares represent the score of each sample site in the first (and largest) global eigenvalue. Color and size differences reflect the genetic differentiation between neighboring sampling sites with large squares representing large absolute values and small squares representing smaller absolute values. Sites with negative values are white, while sites with positive values are black. Thus, a site with a large white square is very differentiated from a site marked by a large black square, but less differentiated from a site marked with a small black square. The most differentiated site in the northern portion of the island is #13, the most likely source of the local eigenvalue. The sites on the northern half of the island (numbers 2, 3, 5, 12, 13, and 14) are quite differentiated from the sites in the southern and central portions of the island. The southern/central sites #4 and #8 were geographically closest to sites in the north and although they produced positive absolute values, the values were smaller, indicating less differentiation than those sites clustered in the center of the island. All subsequent analyses and tests of statistical significance divided animals into two groups: those located in the northern portion of the island with negative global scores and those in the southern/central portion of the island, with positive global scores.

The pairwise Fst [28] between the two pooled clusters comprising the six populations in the north (N = 57) and the eight southern/central populations (N = 176) was 0.011 while Fis [29] was 0.165 in the northern cluster and 0.150 in the southern/central cluster. Observed allele numbers and expected and observed heterozygosities for the two clusters are also shown in Table 1. The northern and southern/central clusters exhibited averages of 5.18±2.26 and 5.92±2.60 alleles per locus, respectively. A paired t-test [30] identified this difference as statistically significant (two-tailed, p<0.001). Average estimates of observed heterozygosity of the northern and southern/central clusters were 0.522 and 0.54, respectively, indicating that the greater number of alleles in the southern/central cluster has led to only marginally greater genotypic variation.

**Figure 4:**
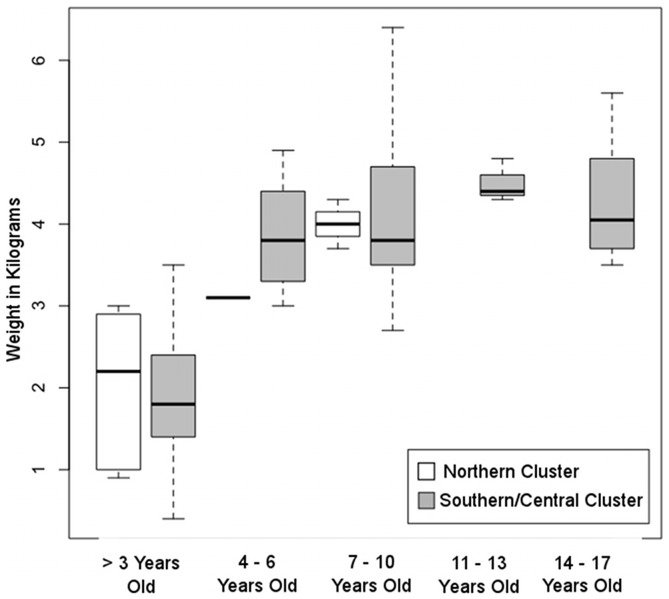
Body weight at capture for each of five age groups. No individuals in the oldest two age groups were sampled from the northern population so only three statistical comparisons could be made. There were no statistically significant differences in body weight between the two groups.

The results of the phenotypic analysis are shown in Figure 4. Although weight at capture was recorded for each of the five age groups (three years old and younger, four to six years old, seven to ten years old, eleven to thirteen years old and fourteen to seventeen years old), none of the animals captured from the northern population fell into the two oldest age groups, precluding statistical comparisons. Comparisons of the three youngest age groups identified no statistically significant difference in weight at capture between the northern and central/southern populations.

## Discussion

Sussman and Tattersall [4] infer that the spread of *M. fascicularis* across Mauritius must have been “explosive”, as no monkeys were mentioned in a list of animals found on Mauritius just eight years before the first description of monkeys on the island in 1606. Such a rapid expansion in the relatively recent past naturally fosters minimal geographic structure. Kondo et al. [10] examined electrophoretic mobility of 32 blood proteins in 201 blood samples from eight regions across south and south central Mauritius. They estimated genetic differentiation in the island population as G_ST_ = 0.0892, with Nei's pairwise genetic distance between sampling sites not exceeding 0.0127; the authors concluded that there was no remarkable genetic differentiation between the sampling sites in south and south central Mauritius. These results are comparable to our comparison of the northern and south/central clusters; (Fst = 0.011), and our PCA could not significantly differentiate any of the sampling sites. In combination with the low mtDNA diversity and MHC homogeneity [2], [17], our results suggest Mauritian animals are very genetically homogenous and lack significant population structure.

Despite the lack of true genetic differentiation, the sites on the northern coast of the island exhibited a greater correlation between geographic and genetic distance than the sampling sites in the south and south central portions of the island, suggesting that the dispersal among animals on the north coast is restricted compared to animals elsewhere. Mauritius is ringed by a low-lying coast that rapidly rises to a central plateau, the highest point of which roughly corresponds to the positions of sampling sites 1, 6, 7, and 10 [31], and extends northward toward the center of the island. However, with a maximum elevation of 600 meters and an average elevation of 300–400 meters, the plateau is likely too low to constitute any barrier to gene flow. In addition, as shown in the connection network (Figure 3), the individuals in the sampling sites along the north coast (3, 13, 5, 14, 2 and 12) exhibit a greater degree of differentiation from their neighbors than those in the south/central region, even though they are located on the coastline with no geographic barriers among them. The Fis values for both the northern and southern/central cluster are positive and nearly identical, indicating an excess of homozygotes (negative Fis values are produced by populations with heterozygosity exceeding random expectation). Excess homozygosity, alternatively, is often a marker of inbreeding. Thus, while there is evidence for non-random mating on Mauritius, the fact that both clusters have very similar Fis values suggests that inbreeding is not the genetic force driving the geographic-genetic correlation in the northern cluster.

Despite anecdotal reports that cynomolgus macaques on the northern end of Mauritius are larger than that elsewhere on the island, our analysis did not support this observation. No statistically significant difference in weight at capture was found between animals through 10 years of age sampled from the northern sites and the southern/central sites. The age at first reproduction in this species is approximately four years [32], suggesting that the range of ages compared extends well past the point at which individuals would have ceased growing. Fooden [1] reported an average weight 3.59 kg for female *M. fascicularis* across the entire species' range, comparable to the values observed for the seven- to ten-year-old age group for both the northern (average female weight at capture = 4.0 kg) and southern/central (average female weight at capture = 4.1 kg) populations. Although both populations are slightly larger than the species average, they are nearly indistinguishable from each other. We conclude that differences in body size are not a factor in the genetic differentiation of *M. fascicularis* on Mauritius – body size differences neither drive nor result from genetic differences.

Founder effect and subsequent rapid expansion have resulted in a paucity of mtDNA, MHC and microsatellite variability in Mauritian, compared to other populations of cynomolgus macaques[26], but these genetic patterns do not differentiate the northern and southern/central clusters. Why, then, is the correlation between geographic and genetic distance greater on the northern coast than elsewhere on the island?

The resolution of this paradox may lie in the history of urbanization and development on the island of Mauritius. With a population of 1.1 million people (590 people/km^2^), Mauritius has one of the greatest population densities of any sovereign territory in the world. Between 1965 and 1986 urban land use increased by over two-fold [33]. During this period, but especially in the 1980's, the tourism industry overtook sugar cane farming as a source of income; currently, over 50 hotels occupy little more than 30 km of coastline, situated largely on the north and northwestern edges of the island [31]. The new food sources resulting from the increased tourism and density of human occupation may have reduced the range size and dispersal distance of monkeys in the north, too recently to be reflected by increased inbreeding, resulting in the genetic partitioning observed by the sPCA. In contrast, monkeys in southern and central Mauritius may still rely predominantly on natural forage and the raiding of sugar cane fields, requiring larger range sizes and increased contact among groups. With an eight-year generation time [32], true genetic differentiation may require either a longer time span or an evolutionary force stronger than home range contraction.

Over 800 square kilometers of the non-urban land in Mauritius (more than 40% of total land area) is dedicated to sugar cane plantations [31]. Sussman and Tattersall [4] and later, Sussman et al. [5] performed one of the few behavioral studies of *M. fascicularis* on Mauritius in 1986 at a site on the western side of the island. They found that the monkeys were largely omnivorous, and while sugar cane made up only eight percent of the diet, it was greatly preferred to more “natural” dietary items. Cynomolgus macaques are known to raid crops throughout Asia, and farmers in Mauritius erect electric fences to keep monkeys out of fields, a strategy that is not completely successful [34].

While making no direct comparison of home range sizes of northern groups versus those elsewhere on the island, Sussmann and Tattersall [4], [5] observed a group in western Mauritius with a home range size of 0.42 km^2^, slightly larger than that reported for other groups of *Macaca fascicularis* throughout Asia. Rodman [35] and Kurland [23] reported home range sizes of 0.32 km^2^ and 0.2 km^2^, respectively, for groups of cynomolgus macaques in the Kutai reserve, Indonesia that participated in crop raiding, although not specifically of sugar cane. Clinical and physiological examinations of animals in southern [36] and western [37] Mauritius found that the animals were adequately nourished, although Tattersall and Sussman [37] observed two individuals with blood glucose levels sufficiently high to suggest pre-diabetes. The larger home range suggests that nutrition comes at a cost, as the animals are required to cover a larger distance than other populations of cynomolgus macaques in order to achieve it.

## Conclusions

Despite broader sampling, the results of this study were concordant with previous genetic investigations of cynomolgus macaques on Mauritus. While significant genetic differentiation among the 14 sampling sites was not found, individuals formed two clusters in the PCA that roughly corresponded to the northern and southern/central portions of the island, albeit with substantial overlap. The Fst between the two groups was low, and calculations of Fis were comparable, suggesting a consistent excess of homozygosity across the island.

A higher correlation between genetic and geographic distance was found in sites on the northern portion of the island compared to the southern/central portion. This divide may be moderately affected by changes in topography, but topography alone does not adequately explain the differentiation between neighboring sites along the uniformly low-lying northern shore. Similarly, no significant difference in body weight at capture was found between animals in the northern and southern/central parts of Mauritius. We hypothesize that the increase in human habitation and tourist infrastructure in northern Mauritius since the 1980s has contributed to, if not created, this effect, as animals supplement their diets with food from anthropogenic sources, reducing both the travel distance required to secure adequate nutrition and contact with neighboring groups.

## Materials and Methods

This study was carried out in strict accordance with the recommendations in the Guide for the Care and Use of Laboratory Animals of the National Institutes of Health and the Weatherall report, “The use of non-human primates in research”. The Institutional Animal Care and Use Committee of the University of California-Davis approved the protocol (#16549). The research described herein is part of larger study in which blood samples for DNA analysis were collected from multiple cynomolgus macaque breeding facilities and analyzed for purposes of genetic management, identification of country of origin, kinship, and phenotype association studies. Only blood samples were used as a DNA source in this study. Blood was collected from anesthetized animals following the standard blood collection procedure at the facility in question, not exceeding 2 ml per kg of body weight.

Whole blood was collected from 233 cynomolgus macaques from 14 different locations on the island of Mauritius, as shown in Figure 1, under the supervision of a veterinarian. Sample sizes as well as the coordinates of the sampling locations are shown in Table 1. Total DNA was extracted from the samples using a guanadinium thiocyanate protocol without a phenol/chloroform step (Froenicke & Sullivan, pers. comm.).

All samples were genotyped at 28 autosomal microsatellite loci, listed in Table 1. These loci have been included in other genetic studies of cynomolgus macaques [25], [26]. For PCR amplification, 6.5 µl of DNA extract was added to each 12.5 µl PCR reaction containing 67 mM Tris Cl (pH 8.8), 16 mM (NH_4_)_2_SO_4_, 0.01% Tween-20, 0.05 mM each dNTP, 0.2 µm each primer, 1.7 mM MgSO_4_, 0.025 units/ml; Invitrogen Platinum Taq. The annealing temperatures and final hold times were independently optimized for each primer pair, but included an initial hold of 94°C for 3 minutes followed by 60 cycles of denaturing at 94°C for 30 seconds, annealing at 56–60°C for 30 seconds, extension at 72°C for 45 seconds and finally a hold at 72°C for 5–60 minutes. All samples were analyzed on an ABI 3130×l genetic analyzer following the manufacturer's protocol and using the GeneScan™ LIZ size standard to assign genotypes. No individual had more than 5% missing data.

Allele number per locus as well as observed and expected heterozygosity were calculated with the adegenet package 1.3–1 for R [38]. The same program was used to perform both a principal component analysis (PCA) and a spatial principal component analysis (sPCA) with a distance network built via the relative neighbors method [39]. Real distance between sampling coordinates was calculated with Google Earth (earth.google.com).

Upon capture, each animal was weighed and age was estimated from dentition and body condition. Individuals were categorized by age at capture into the following groups: three years old or less, four to six years, seven to ten years, eleven to thirteen years, and fourteen to seventeen years. Age could not be accurately determined for 110 of the individuals. To avoid biasing the comparison of body weight, ten males were removed from the analysis. One of the males was from the northern population (site 13) while the remaining eight males were from the southern/central population (three from site 4, two each from sites 6 and 11, and one each from sites 8 and 9). Of the remaining 113 females, 11 were sampled from the northern population and 101 were sampled from the southern/central population. The distribution of weight at capture for each age group was tested for normality using the Shapiro-Wilk test [40] and all were found to significantly deviate from a normal distribution. Individuals from the northern and southern/central populations were compared separately for each age group using the Wilcoxon rank sum test [41]. All statistical comparisons were performed in the R environment [42]. Only three comparisons could be performed, as there were no individuals captured from the northern population in the 11 to 13 or 14 to 17-year-old age groups.

## Supporting Information

Table S1 Observed and expected heterozygosity values for each of the sampling sites.
(XLSX)
